# Unexpected Sex Differences in the Relationship of Sacroiliac Joint and Lumbar Spine Degeneration

**DOI:** 10.3390/diagnostics12020275

**Published:** 2022-01-21

**Authors:** Maximilian Muellner, Virginie Kreutzinger, Luis Becker, Torsten Diekhoff, Matthias Pumberger, Friederike Schömig, Mark Heyland, Katharina Ziegeler

**Affiliations:** 1 Center for Musculoskeletal Surgery, Charité—University Medicine Berlin, 10117 Berlin, Germany; maximilian.muellner@charite.de (M.M.); luis-alexander.becker@charite.de (L.B.); matthias.pumberger@charite.de (M.P.); friederike.schoemig@charite.de (F.S.); 2 Department of Radiology, Vivantes Klinikum im Friedrichshain, 10249 Berlin, Germany; v.kreutzinger@gmail.com; 3 Department of Radiology, Charité—Universitätsmedizin Berlin, 10117 Berlin, Germany; torsten.diekhoff@charite.de; 4 Berlin Institute of Health at Charité—Universitätsmedizin Berlin, Julius Wolff Institute, 13353 Berlin, Germany; mark.heyland@bih-charite.de

**Keywords:** sacroiliac joint, lumbar spine, degeneration, pelvic incidence, sacral table angle, pelvic radius

## Abstract

The relationship between degenerative changes of the sacroiliac joints and the lumbar spine on CT has not been studied yet. The aim of this analysis is to determine the nature of their association as well as the influence of fixed anatomical spinopelvic parameters on sacroiliac joint degeneration. For this institutional review-board-approved investigation, imaging datasets as well as electronic medical records of 719 patients without back pain from the clinical routine of our department of radiology were included. Age, sex, weight category (slim, normal, obese), parity in women and indication for imaging were noted for all patients. The presence of degenerative lesions of the lumbar spine (disc degeneration, endplate degeneration, spondylophytes, and facet joint osteoarthritis) was noted separately at each lumbar segment (L1 to L5). Sacroiliac joints were assessed for sclerosis and osteophytes. Fixed anatomical spinopelvic parameters were measured: pelvic radius = PR; pelvic incidence = PI; sacral table angle = STA. Correlation as well as regression analyses were performed; data were analyzed for males and females separately. PI increased significantly with age in both women and men, while STA decreased and PR remained constant; neither of them was associated with SIJ degeneration. SIJ degeneration correlated with disc degeneration (tau = 0.331; *p* < 0.001), spondylophytes (tau = 0.397; *p* < 0.001), and facet joint degeneration (tau = 0.310; *p* < 0.001) in men, but with no parameter of spinal degeneration in women. Lumbar spinal degeneration increased the risk of sacroiliac joint degeneration in men significantly (OR 7.2; 95%CI 2.8–19.0), but it was not a significant covariable in women. Fixed spinopelvic parameters have little impact on sacroiliac joint degeneration. The degeneration of the sacroiliac joints and the lumbar spine appear to be parallel processes in men, but are largely unrelated in women.

## 1. Introduction

The sacroiliac joint (SIJ) is one of the most important mechanical axes of the human body, responsible for the transmission of force from the lower extremities to the lumbar spine [1]. The joint itself has a limited range of motion with nutation and in- or outflare movement [2]. It is believed to function mainly as a shock absorber, especially during bipedal walking [3]. Over the course of a lifetime, nonspecific low back pain is experienced by up to 80% of the population and accounts for significant medical costs. The degenerative or mechanical disease of the SIJ, which may be the root cause of low back pain in as many as 30% of patients [4,5], is still challenging to assess clinically [6]. One of the factors that may have an impact on biomechanical stress to the joint is the overall pelvic angulation or shape, which may be described quantitatively by the pelvic radius (PR), pelvic incidence (PI) and sacral table angle (STA). Especially, the pelvic incidence is of great importance in the restoration of the sagittal profile in adult spinal deformity surgery. It is the key parameter for the assessment of the sagittal profile by using the Global Alignment and Proportion (GAP) score, which predicts mechanical complications in patients undergoing spinal deformity correction [7].

Furthermore, the difference between pelvic incidence and lumbar lordosis in the SRS-Schwab classification is an important parameter for the prognosis of health-related quality of life in deformity surgery [8]. The degeneration of the SIJ is affected by the spino-pelvic parameters. A recent study by Kwon et al. [9] found a positive correlation between pelvic incidence and SIJ degeneration in adult spinal deformity, but not in controls with normal sagittal balance. Another aspect that has received very little attention from the scientific community is the association of degeneration of the lumbar spine and the SIJ. However, from a clinical perspective, these co-existing pathologies are highly relevant to patients suffering from one or the other degenerative pain condition.

Although large-scale population-based data on gender distribution of SIJ pain syndromes is currently missing, there is evidence that females are more commonly afflicted, with proportions of females of over 70% [10,11] in different clinical trials of SIJ dysfunction. Previous investigations of the patient cohort presented in this analysis revealed sex differences in the overall spatial distribution of degenerative lesions across the SIJ [12] and a propensity of females to exhibit joint form anomalies, which are in turn associated with SIJ disease [13,14]. These factors, together with the well-established gender differences in specific joint biomechanics [15], underline the need for a sex-disaggregated analysis of degenerative SIJ changes [16].

The aim of this investigation is to investigate the impact of pelvic angulation, described by pelvic radius, pelvic incidence, and sacral table angle on the distribution of degenerative lesions in a large study cohort without low back pain. Furthermore, the correlation with degenerative findings of the lumbar spine is evaluated, with special focus on sex-specific differences.

## 2. Materials and Methods

### 2.1. Patients and Exclusion Criteria

**Figure 1:**
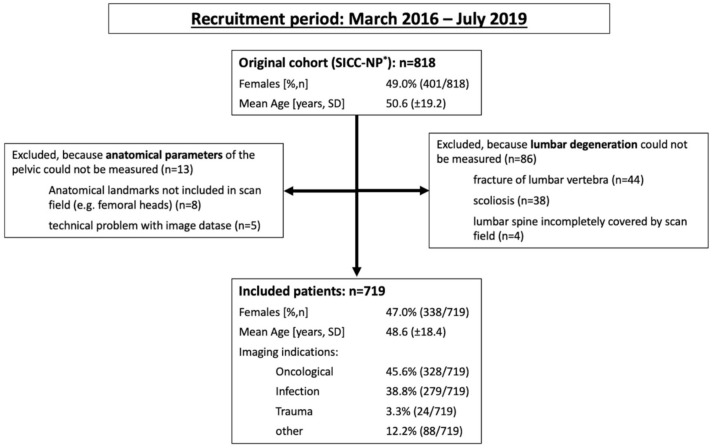
Patient flow. SICC-NP* = sacroiliac changes in the normal population study [12].

This study was approved by the institutional review board of the Charité—Universitätsmedizin Berlin (EA1/300/19) and conducted in accordance with local legislation and ethical standards as well as the Declaration of Helsinki. The investigation is a secondary analysis of a larger cohort of patients from the normal population without low back pain—detailed information on this cohort can be found in a separate publication [12]. A flow diagram of the assembly process of the study participants is given as Figure 1. Electronic patient records were searched for age, gender, nutritional status (slim, normal, obese), and parity (in women), as well as indication for the CT examination (oncological staging, search for infectious focus, trauma, bleeding, among others). To rule out identification of individual patients, all CT and demographic patient data were anonymized using a dedicated software.

### 2.2. Scoring of Degenerative Lesions

**Figure 2:**
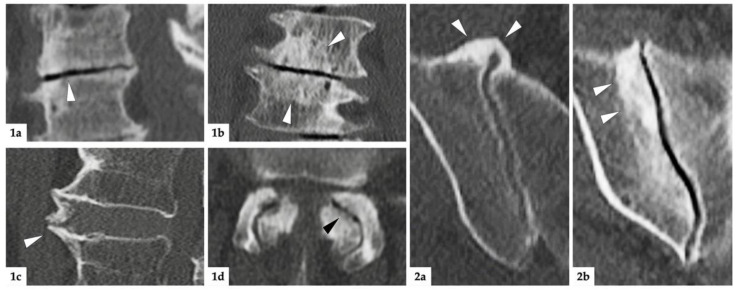
Imaging examples. (**1**): Degenerative lesions of the lumbar spine; (**1a**) = sagittal reconstruction: white arrowhead marks discal vacuum phenomenon and narrowing of intervertebral space; (**1b**) = coronal reconstruction: white arrowheads mark sclerosis of endplates; (**1c**) = sagittal reconstruction: white arrowhead indicates spondylophyte; (**1d**) = axial reconstruction: black arrowhead marks intraarticular vacuum phenomenon. Additionally, note the extensive sclerosis and joint space irregularities from OA of the facet joints. (**2**): degenerative lesions of the SIJ; (**2a**) = axial reconstruction: ventrally located, bridging osteophytes of the right SIJ marked with white arrowheads; (**2b**) = oblique-coronal reconstruction: extensive sclerosis around the joint (white arrowheads).

A radiologist with 5 years of clinical experience and expertise in musculoskeletal imaging (K.Z.) scored all images in random order, blinded to clinical data. Images were read using a dedicated software (Horos v3.3.6, The Horos Project, public license). SIJ degeneration was assessed as periarticular sclerosis of at least 2 mm width and/or osteophytes at the ventral or dorsal aspect of the joint. A more detailed description of this assessment has been published in detail elsewhere [12]. The degeneration of the lumbar spine was assessed separately for spinal segments L1/L2 through L5/S1; in each segment, the presence or absence of disc degeneration (defined as: marked narrowing of the intervertebral space and/or vacuum phenomenon of the disc), endplate degeneration (defined as: marked sclerosis of >3 mm), spondylophyte formation, and osteoarthritis (OA) of the facet joints (defined as: narrowing of the joint space and presence of osteophytes around the facet joints) was noted. Imaging examples for all instances of degeneration are provided in Figure 2. To assess inter-reader agreement, a random sample of 40 patients was assessed by a junior radiologist (V.K.), applying the same scoring system.

### 2.3. Anatomical Measurements

**Figure 3:**
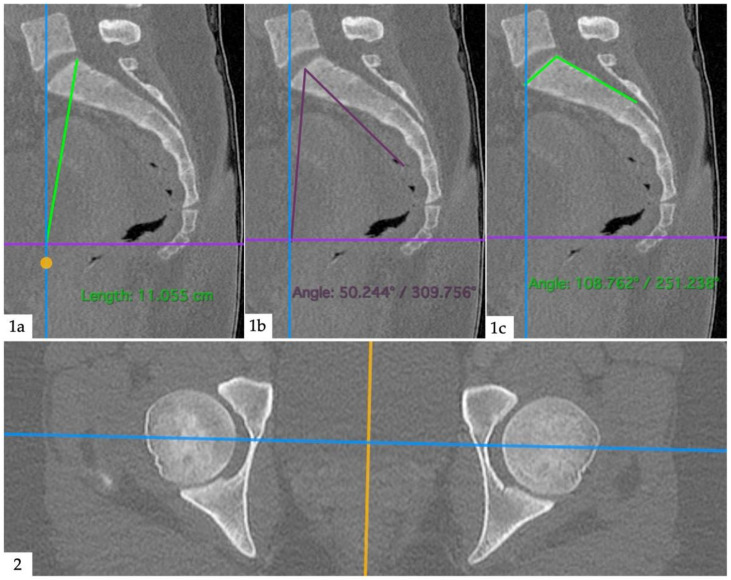
Anatomical measurements. (**1a**): Pelvic radius; distance between posterior–superior corner of the first sacral vertebra and the center of a line between the femoral heads (intersection between blue and violet lines). (**1b**): Pelvic incidence; angle between a line from the center of a line between the two femoral heads and the center of the sacral plateau and a line perpendicular to the sacral plateau. (**1c**): Sacral table angle; angle between the posterior wall of the sacrum and the sacral plateau. (**2**): Second plane (axial) to aid orientation.

Anatomical measurements were performed by an orthopedic surgeon (M.M.) with the same software (Horos v3.3.6, The Horos Project, public license), using dynamic multiplanar reconstructions and triangulation. Pelvic radius (PR) was defined as the distance between the posterior–superior corner of the first sacral vertebra and the center of a line between the two femoral heads. Pelvic incidence (PI) was defined as an angle formed between a line from the center of a line between the two femoral heads and the center of the sacral plateau and a line perpendicular to the sacral plateau. Sacral table angle (STA) was defined as an angle between the posterior wall of the sacrum and the sacral plateau. An example image of the measurements is given as Figure 3. A junior radiologist (V.K.) performed measurements on 40 randomly selected sample patients to assess inter-reader agreement.

### 2.4. Statistical Analysis

Statistical analysis was carried out using SPSS version 27 (IBM Corporation, New York, NY, USA). For the SIJ, positivity for degeneration was defined as marked sclerosis in at least one joint region or prominent osteophytes in any location were considered positive for SIJ degeneration. Means and standard deviations (SD) of anatomical measurements and frequency of degenerative findings of the lumbar spine and SIJ were compared between male and female patients for each age group separately using *t*-tests and Chi2 tests, respectively. Inter-reader agreement was assessed using intra-class correlation coefficients (ICC) with a two-way mixed model and reported according to Koo et al. [17]. To confirm suspected age trends, the Jonckheere–Terpstra test was applied. Correlations between SIJ degeneration and clinical and anatomical parameters, as well as degeneration of the lumbar spine, were investigated using Kendall-tau-b. Furthermore, the impact of each of these factors on SIJ degeneration was investigated using binomial logistic regression with the presence of lumbar spinal degeneration, nutritional status, age, PR, PI, STA and parity as covariables, again separately for both sexes. To avoid inflation of the alpha error in multiple correlation analyses (*n* = 50), a Bonferroni correction was used (*n* = 50), resulting in an adjusted *p*-value of *p* < 0.001 for this set of results. For all other tests, a significance level of *p* < 0.05 was assumed.

## 3. Results

### 3.1. Patients

After the application of the exclusion criteria, a total of 719 patients were included in this investigation; their clinical characteristics are given in Figure 1. Frequencies of obesity did not differ between the sexes, with 40.4% of affected males vs. 39.6% (*p* = 0.405) affected females. Of the included females, 40.8% had never given birth, 44.4% had given birth to one or two children, and 14.8% had given birth to three or more children.

### 3.2. Anatomical Measurements per Age Group

A summary of anatomical measurements per age group is given in Table 1. The Jonckheere–Terpstra test showed that the observed increase in PI in males reflects a significant age trend (*p* = 0.025), as do the increase in PI (*p* = 0.015) and the decrease in STA (*p* = 0.002) in females.

### 3.3. Degenerative Findings per Age Group

The distribution and relative frequencies of degenerative lesions of the sacroiliac joints in this study cohort have been described in detail elsewhere [12]. The degenerative findings of the lumbar spine are given both per age group and per segment in Table 2 and Table 3, respectively. Disc degeneration did not differ between the sexes, with a steady increase in prevalence across age groups. Endplate degeneration was more common in older females than males, but it did not differ in overall prevalence. Spondylophytes were more overall more common in males—the difference was most pronounced in the age group of 35–44 years. Regarding the lumbar segments that are most affected, males exhibited a propensity for spondylophytes of the segments L2 and L3.

### 3.4. Association of Degeneration, Anatomical Measurements and Clinical Factors

**Figure 4:**
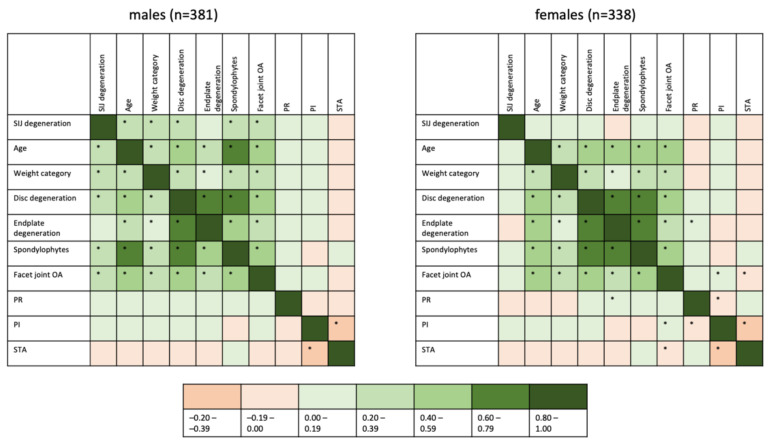
Heatmap of correlations between degeneration, clinical and anatomical factors. Correlation coefficients were derived from Kendall-tau-b analyses. Significant (*p* < 0.001) correlations are marked with an asterisk within the box. As most associations were positive, an asymmetrical color scale was used for ease of interpretation.

Correlations between SIJ degeneration, age, weight, disc degeneration, endplate degeneration, spondylophytes, facet joint OA, PR, PI and STA were investigated using Kendall-tau-b—a graphical representation of the resulting correlation coefficients is given as Figure 4. This graphic reveals differences in the association patterns between males and females, with a slightly stronger inter-relatedness of different forms of lumbar degeneration in females than in males, yet a lack of associations of degenerative lesions of the lumbar spine and SIJ degeneration in females. Some inter-relatedness of fixed anatomical markers is observed (e.g., a weak-to-moderate negative correlation between PI and STA in both sexes), but no significant correlation with either SIJ or lumbar spine degeneration is seen.

Logistic regression analyses for SIJ degeneration yielded a much higher model accuracy for men with a Nagelkerke’s R^2^ of 0.262 vs. only 0.046 in females. In males, the strongest association was shown for overall lumbar spinal degeneration with an OR of 7.2 (95%CI 2.8–19.0; *p* < 0.001) followed by age group with an OR of 1.2 (95%CI 1.0–1.4; *p* = 0.012), while all other factors missed statistical significance. In females, only age group was a significant covariable with an OR of 1.2 (95%CI 1.0–1.4; *p* = 0.043).

### 3.5. Inter-Reader Reliability

Inter-reader agreement, expressed by ICCs, was good for disc degeneration (0.810; 95%CI 0.669–0.895; *p* < 0.001), excellent for spondylophytes (0.921; 95%CI 0.856–0.957; *p* < 0.001), and moderate for both endplate degeneration (0.552; 95%CI 0.293–0.735; *p* < 0.001) and facet joint OA (0.622; 95%CI 0.388–0.781; *p* < 0.001). Agreement regarding anatomical parameters of the pelvis was good to excellent with ICCs of 0.940 (95%CI 0.887–0.968; *p* < 0.001), 0.968 (95%CI 0.940–0.983; *p* < 0.001), and 0.893 (95%CI 0.798–0.944; *p* < 0.001) for PR, PI, and STA, respectively. Data on agreement regarding SIJ degeneration were published separately [12].

## 4. Discussion

To the best of our knowledge, this is the first investigation of the association of quantitative anatomical parameters of the pelvis and degeneration of the lumbar spine with sacroiliac joint degeneration in a large cohort of the normal population. Surprisingly, although the association between degenerative findings of the lumbar spine and the SIJ was strong in men, no such association could be demonstrated in women.

In males, the close relationship of lumbar spinal degeneration and SIJ degeneration is mirrored in both the 7.2-fold risk of observing SIJ degeneration when lumbar spinal degeneration is present and the correlation of SIJ degeneration with disc degeneration (tau = 0.331), spondylophytes (tau = 0.397) and facet joint OA (tau = 0.310). Postoperative SIJ pain is one of the most common complaints after spondylodesis [18]. The possibility of concomitant SIJ related pain with degenerative conditions of the lumbar spine should therefore be clinically explored preoperatively [19], as operative fusion of the SIJ [20] could be achieved by spino-pelvic anchoring in the same procedure [21]. In females, no association of SIJ degeneration and anatomical parameters or degeneration of the lumbar spine could be shown. This underlines the special biomechanical role of the female SIJ, which has been demonstrated by Joukar et al. [15] to be exposed to more stresses and loads than the male joint in finite element models. In our investigation, PR remained constant with age, while PI showed an increase and STA a decrease with age. These findings are in line with those of Kwon et al. who found a correlation between PI and age [9], yet somewhat contradict those of Baker et al. [22], who investigated a smaller cohort of patients without spinal disease. Based on our results, the surgical restoration of the sagittal profile in adult spinal deformity with increasing age should aim for increased lumbar lordosis in line with increasing PI to reduce the risk of persistent imbalance and worse patient outcome [7,8]. In our study population, PI, STA, and PR were not associated with SIJ degeneration. Furthermore, we did not find a significant correlation between either of these parameters and degenerative lesions of the lumbar spine, which somewhat differs from the findings of Strube et al. [23]. This disparity is best explained by the fact that we investigated patients without known back pain and used CT rather than radiography.

There are some limitations to this investigation that warrant further discussion. Only computed tomography images were used to assess lumbar spine degeneration, and this may have led to an underestimation of disc degeneration, as only advanced findings are reliably captured on CT as opposed to MRI [24,25,26]. Furthermore, as we only included scans acquired in a supine position, quantitative parameters of spinopelvic orientation, such as sagittal balance, could not be measured. This is an important limitation, because recent studies have underlined the importance of lumbar lordosis and sagittal vertical axis for degenerative lesions of the SIJ [9]. Furthermore, clinical and epidemiological patient data, such as occupational or recreational physical activity, were not available due to the retrospective setting of this investigation. Lastly, there is controversy among experts, whether degenerative SIJ lesions in CT are an appropriate surrogate marker for biomechanical stress and joint disease [27], as significant proportions of asymptomatic patients may exhibit such findings [12,28]. Finite element analyses [29] may be more appropriate to illicit the effect of joint angulation on the distribution of mechanical load.

## 5. Conclusions

The degeneration of the SIJ and the lumbar spine in patients without reported low back pain appear to be parallel processes in men and largely unrelated in women. Furthermore, fixed anatomical parameters alone have only a minor influence on degenerative lesions of the SIJ in the normal population. Further studies are needed for a better understanding of the cofactors of SIJ degeneration and mechanical joint disease with a continued focus on sex differences.

## Figures and Tables

**Table 1:** Anatomical measurements per age group. Measurements are given as means and standard deviations (SD). Significantly larger values compared to opposite sex are printed bold and marked with an asterisk (*); *p*-values were derived from *t*-tests.

Age Group	*n*	Pelvic Radius. Mean and SD (cm)		*p*	Pelvic Incidence. Mean and SD (Degrees)		*p*	Sacral Table Angle. Mean and SD (Degrees)		*p*
		Male	Female		Male	Female		Male	Female	
<25	60	11.83 (0.98)	11.66 (0.91)	0.508	46.82 (11.88)	44.58 (10.98)	0.460	102.77 (5.70)	105.78 * (5.48)	0.044
25–34	128	11.89 (0.83)	11.94 (0.85)	0.766	49.94 (11.52)	48.12 (10.86)	0.361	101.45 (5.49)	102.56 (6.59)	0.316
35–44	116	11.96 (0.77)	11.88 (0.78)	0.589	47.47 (8.92)	51.16 * (11.02)	0.046	101.88 (6.18)	102.59 (5.62)	0.523
45–54	120	11.76 (1.02)	11.69 (0.77)	0.671	49.92 (10.44)	53.74 (8.81)	0.050	101.20 (5.84)	102.87 (6.02)	0.126
55–64	112	11.80 (0.91)	11.81 (0.80)	0.932	50.10 (9.94)	54.17 * (8.81)	0.026	101.73 (5.49)	102.62 (5.53)	0.396
65–74	98	12.02 (0.79)	11.85 (0.73)	0.280	49.48 (9.43)	50.64 (9.09)	0.540	100.48 (5.43)	101.86 (4.79)	0.190
≥75	85	11.79 (0.90)	11.78 (0.87)	0.929	51.87 (10.50)	51.14 (10.50)	0.452	101.31 (5.73)	100.57 (5.97)	0.372
Total	719	11.87 (0.88)	11.82 (0.81)	0.403	49.45 (10.31)	50.82 (10.58)	0.079	101.49 (5.69)	102.55 * (5.87)	0.014

**Table 2:** Frequency of degenerative lesions per age group. Relative and absolute frequencies. Significantly higher frequencies compared to opposite sex are printed bold and marked with an asterisk (*); *p*-values were derived from Chi^2^ test.

Age Group (Years)	Disc (%,*n*)		Endplate (%,*n*)		Spondylophyte (%,*n*)		Facet Joint (%,*n*)	
	Male	Female	Male	Female	Male	Female	Male	Female
<25	0.0% (0/35)	0.0% (0/25)	0.0% (0/35)	0.0% (0/25)	2.9% (1/35)	0.0% (0/25)	2.9% (1/35)	0.0% (0/25)
25–34	6.3% (4/63)	15.4% (10/65)	4.8% (3/63)	7.7% (5/65)	6.3% (4/63)	7.7% (5/65)	15.9% (10/63)	12.3% (8/65)
35–44	29.5% (18/61)	29.1% (16/55)	11.5% (7/61)	7.3% (4/55)	**42.6%** (26/61) *	20.0% (11/55)	24.6% (15/61)	**49.1%** (27/55) *
45–54	42.4% (25/59)	45.9% (28/61)	30.5% (18/59)	23.0% (14/61)	71.2% (42/59)	57.4% (35/61)	55.9% (33/59)	67.2% (41/61)
55–64	68.9% (42/61)	70.6% (36/51)	39.3% (24/61)	47.1% (24/51)	86.9% (53/61)	76.5% (39/51)	75.4% (46/61)	86.3% (44/51)
65–74	81.8% (45/55)	88.4% (38/43)	50.9% (28/55)	**76.4%** (33/43) *	98.2% (54/55)	97.7% (42/43)	94.5% (52/55)	95.3% (41/43)
≥75	85.1% (40/47)	89.5% (34/38)	59.6% (28/47)	**81.6%** (31/38) *	100.0% (47/47)	97.7% (36/38)	95.7% (45/47)	97.4% (37/38)
**Total**	45.7% (174/381)	47.9% (162/338)	28.3% (108/381)	32.8% (111/338)	**59.6%** (227/381) *	49.7% (168/338)	53.0% (202/381)	58.6% (198/338)

**Table 3:** Frequency of degenerative lesions per segment. Relative and absolute frequencies. Significantly higher frequencies compared to opposite sex are printed bold and marked with an asterisk (*); *p*-values were derived from Chi^2^ test.

Age Group (Years)	Disc (%,*n*)		Endplate (%,*n*)		Spondylophytes (%,*n*)		Facet Joint (%,*n*)	
	Male	Female	Male	Female	Male	Female	Male	Female
L1	20.7% (79/381)	23.2% (78/336)	5.5% (21/381)	5.4% (18/336)	32.0% (122/381)	25.9% (87/336)	24.4% (93/381)	21.7% (73/336)
L2	17.1% (65//381)	19.3% (65/336)	4.8% (3/381)	5.4% (21/336)	38.8% (148/381) *	30.1% (101/336)	29.7% (113/381)	28.6% (96/336)
L3	14.7% (56/381)	19.6% (66/336)	4.2% (16/381)	8.0% (27/336) *	41.7% (159/381) *	31.8% (107/336)	35.2% (134/381)	37.8% (127/336)
L4	17.3% (66/381)	22.0% (74/336)	8.9% (34/381)	12.2% (41/336)	37.5% (143/381)	31.5% (106/336)	42.0% (160/381)	47.9% (161/336)
L5	31.0% (118/381)	37.2% (125/336)	18.4% (70/381)	23.2% (78/336)	38.1% (145/381)	36.6% (123/336)	49.9% (190/381)	56.3% (189/336)

## Data Availability

The datasets generated during and/or analyzed during the current study are available from the corresponding author on reasonable request.

## References

[1] Kiapour A., Joukar A., Elgafy H., Erbulut D.U., Agarwal A.K., Goel V.K. (2020). Biomechanics of the Sacroiliac Joint: Anatomy, Function, Biomechanics, Sexual Dimorphism, and Causes of Pain. Int. J. Spine Surg..

[2] Vleeming A., Schuenke M.D., Masi A.T., Carreiro J.E., Danneels L., Willard F.H. (2012). The sacroiliac joint: An overview of its anatomy, function and potential clinical implications. J. Anat..

[3] Toyohara R., Kurosawa D., Hammer N., Werner M., Honda K., Sekiguchi Y., Izumi S.I., Murakami E., Ozawa H., Ohashi T. (2020). Finite element analysis of load transition on sacroiliac joint during bipedal walking. Sci. Rep..

[4] Schwarzer A.C., Aprill C.N., Bogduk N. (1995). The sacroiliac joint in chronic low back pain. Spine.

[5] Sembrano J.N., Polly D.W. (2009). How often is low back pain not coming from the back?. Spine.

[6] Ou-Yang D.C., York P.J., Kleck C.J., Patel V.V. (2017). Diagnosis and Management of Sacroiliac Joint Dysfunction. J. Bone Jt. Surg. Am..

[7] Yilgor C., Sogunmez N., Boissiere L., Yavuz Y., Obeid I., Kleinstuck F., Perez-Grueso F.J.S., Acaroglu E., Haddad S., Mannion A.F. (2017). Global Alignment and Proportion (GAP) Score: Development and Validation of a New Method of Analyzing Spinopelvic Alignment to Predict Mechanical Complications After Adult Spinal Deformity Surgery. J. Bone Jt. Surg. Am..

[8] Schwab F., Ungar B., Blondel B., Buchowski J., Coe J., Deinlein D., DeWald C., Mehdian H., Shaffrey C., Tribus C. (2012). Scoliosis Research Society-Schwab adult spinal deformity classification: A validation study. Spine.

[9] Kwon B.T., Kim H.J., Yang H.J., Park S.M., Chang B.S., Yeom J.S. (2020). Comparison of sacroiliac joint degeneration between patients with sagittal imbalance and lumbar spinal stenosis. Eur. Spine J..

[10] Dengler J., Kools D., Pflugmacher R., Gasbarrini A., Prestamburgo D., Gaetani P., Cher D., Van Eeckhoven E., Annertz M., Sturesson B. (2019). Randomized Trial of Sacroiliac Joint Arthrodesis Compared with Conservative Management for Chronic Low Back Pain Attributed to the Sacroiliac Joint. J. Bone Jt. Surg. Am..

[11] Telli H., Telli S., Topal M. (2018). The Validity and Reliability of Provocation Tests in the Diagnosis of Sacroiliac Joint Dysfunction. Pain Phys..

[12] Ziegeler K., Kreutzinger V., Diekhoff T., Roehle R., Poddubnyy D., Pumberger M., Hamm B., Hermann K.G.A. (2021). Impact of age, sex, and joint form on degenerative lesions of the sacroiliac joints on CT in the normal population. Sci. Rep..

[13] Ziegeler K., Hermann K.G.A., Diekhoff T. (2021). Anatomical Joint Form Variation in Sacroiliac Joint Disease: Current Concepts and New Perspectives. Curr. Rheumatol. Rep..

[14] Ziegeler K., Kreutzinger V., Proft F., Poddubnyy D., Hermann K.G.A., Diekhoff T. (2021). Joint anatomy in axial spondyloarthritis: Strong associations between sacroiliac joint form variation and symptomatic disease. Rheumatology.

[15] Joukar A., Shah A., Kiapour A., Vosoughi A.S., Duhon B., Agarwal A.K., Elgafy H., Ebraheim N., Goel V.K. (2018). Sex Specific Sacroiliac Joint Biomechanics During Standing Upright: A Finite Element Study. Spine.

[16] Heidari S., Babor T.F., De Castro P., Tort S., Curno M. (2016). Sex and Gender Equity in Research: Rationale for the SAGER guidelines and recommended use. Res. Integr. Peer Rev..

[17] Koo T.K., Li M.Y. (2016). A Guideline of Selecting and Reporting Intraclass Correlation Coefficients for Reliability Research. J. Chiropr. Med..

[18] Tonosu J., Kurosawa D., Nishi T., Ito K., Morimoto D., Musha Y., Ozawa H., Murakami E. (2019). The association between sacroiliac joint-related pain following lumbar spine surgery and spinopelvic parameters: A prospective multicenter study. Eur. Spine J..

[19] Telli H., Huner B., Kuru O. (2020). Determination of the Prevalence From Clinical Diagnosis of Sacroiliac Joint Dysfunction in Patients With Lumbar Disc Hernia and an Evaluation of the Effect of This Combination on Pain and Quality of Life. Spine.

[20] Lorio M., Kube R., Araghi A. (2020). International Society for the Advancement of Spine Surgery Policy 2020 Update-Minimally Invasive Surgical Sacroiliac Joint Fusion (for Chronic Sacroiliac Joint Pain): Coverage Indications, Limitations, and Medical Necessity. Int. J. Spine Surg..

[21] Baria D., Lindsey R.W., Milne E.L., Kaimrajh D.N., Latta L.L. (2020). Effects of Lumbosacral Arthrodesis on the Biomechanics of the Sacroiliac Joint. JBJS Open Access.

[22] Baker J.F., Don A.S., Robertson P.A. (2020). Pelvic Incidence: Computed Tomography Study Evaluating Correlation with Sagittal Sacropelvic Parameters. Clin. Anat..

[23] Strube P., Pumberger M., Sonnow L., Zippelius T., Nowack D., Zahn R.K., Putzier M. (2018). Association Between Lumbar Spinal Degeneration and Anatomic Pelvic Parameters. Clin. Spine Surg..

[24] Quattrocchi C.C., Alexandre A.M., Della Pepa G.M., Altavilla R., Zobel B.B. (2011). Modic changes: Anatomy, pathophysiology and clinical correlation. Acta Neurochir. Suppl..

[25] Kos N., Gradisnik L., Velnar T. (2019). A Brief Review of the Degenerative Intervertebral Disc Disease. Med. Arch..

[26] Colosimo C., Gaudino S., Alexandre A.M. (2011). Imaging in degenerative spine pathology. Acta Neurochir. Suppl..

[27] Backlund J., Clewett Dahl E., Skorpil M. (2017). Is CT indicated in diagnosing sacroiliac joint degeneration?. Clin. Radiol..

[28] Eno J.J., Boone C.R., Bellino M.J., Bishop J.A. (2015). The prevalence of sacroiliac joint degeneration in asymptomatic adults. J. Bone Jt. Surg. Am..

[29] Ivanov A.A., Kiapour A., Ebraheim N.A., Goel V. (2009). Lumbar fusion leads to increases in angular motion and stress across sacroiliac joint: A finite element study. Spine.

